# Antifungal effects of essential oils on the preservation of historical monuments: A sustainable approach

**DOI:** 10.1371/journal.pone.0338615

**Published:** 2026-01-05

**Authors:** Hatice Yıldız Acar, Hacer Bakır Sert

**Affiliations:** Akdeniz University, Institute of Science, Antalya, Turkey; National Institute of Agricultural Research - INRA, MOROCCO

## Abstract

This study investigates the antifungal properties of essential oils derived from plants in the Lamiaceae family for the preservation of historical structures affected by black microcolonial fungi in the Ancient City of Side, located in Southern Anatolia. Black microfungi, known for their role in the biodeterioration of cultural heritage, were isolated from corroded historical structures. The study specifically focused on essential oils extracted from *Origanum majorana* L., *Origanum minutiflorum* O. Schwarz & P.H. Davis, and *Mentha longifolia* L. subsp. *typhoides* (Brig.) Harley. The antifungal activity of these essential oils was evaluated under in vitro conditions at three concentrations (0.1%, 1%, and 3%). Microfungal cell counts were performed before and after the application of the essential oils. The results demonstrated a concentration-dependent antifungal effect, with higher concentrations (3%) showing the most significant inhibition of fungal growth. Essential oils from *O. majorana*, *O. minutiflorum*, and *M. longifolia* effectively suppressed the growth of *Coniosporium*, *Sarcinomyces*, and *Phaeococcomyces* species, which are major contributors to the biodeterioration of historical structures in the study area. These findings highlight the potential of essential oils as eco-friendly and sustainable antifungal agents for the conservation of cultural heritage. By mitigating black microfungal colonization, these natural compounds offer a promising alternative to synthetic biocides, aligning with the growing demand for environmentally friendly preservation methods. Further research is recommended to explore their long-term efficacy and practical applications in the field of cultural heritage conservation.

## 1. Introduction

Historical buildings and artifacts are invaluable cultural assets that preserve the legacy of past civilizations. However, these structures face constant threats from various environmental factors, including air pollution, temperature fluctuations, humidity, wind, heavy rain, and biological agents such as fungi. Along with fungi, other major biological agents responsible for the deterioration of historical monuments include cyanobacteria, algae, lichens, and bacteria. These microorganisms often co-colonize stone surfaces, leading to both structural and aesthetic damage [1–6], including cracks, exfoliation, discoloration, and surface abrasions, which diminish both the aesthetic appeal and structural integrity of historical monuments over time [7–10].

Efforts to mitigate these deteriorative effects have focused on developing preservation methods that are both effective and safe for materials such as marble, sandstone, and limestone. Chemical treatments, including naturally derived compounds such as plant essential oils, have been widely used; however, these chemical applications often pose risks to the structural integrity of these materials and the surrounding environment. In recent years, there has been growing interest in sustainable chemical alternatives, particularly plant-derived essential oils, which are considered safer than traditional synthetic chemicals [11–17].

Plants and their derivatives have long been utilized across various industries, including medicine, perfumery, cosmetics, and food production, for their diverse applications ranging from therapeutic uses to culinary flavoring [18–21]. Among these, the Lamiaceae family is particularly notable for its aromatic and medicinal properties. This family, which is abundant in the Mediterranean region, contains plants with active compounds whose composition is influenced by genetic factors, climate, environmental conditions, and cultural practices [22,23].

Essential oils derived from Lamiaceae plants exhibit potent antibacterial, antifungal, and antiseptic properties. Their antifungal activity is primarily attributed to their ability to disrupt fungal membranes through interactions with cell-bound lipophilic compounds, leading to alterations in membrane structure and function. This disruption inhibits fungal growth and metabolic activities [14,24,25]. Additionally, essential oils interfere with enzymatic reactions within microbial cells, reduce nutrient uptake, and suppress fungal proliferation [26–29]. In restoration applications, it is important to consider not only the efficacy and toxicological properties of biocides, but also their potential interactions with the substrate. The literature recommends standardized approaches and comparative analyses to assess the effects of biocides on stone materials [30,31]

Microfungi, particularly black microcolonial fungi, pose significant threats to historical artifacts. These fungi mechanically perforate stone surfaces and cause structural deterioration through acid secretion and physical expansion within stone matrices [32,33]. Once adhered to stone surfaces, fungal hyphae penetrate crystal structures, forming colonies within cracks and voids. This process generates internal pressures that lead to stone cracking, fragmentation, and eventual structural loss, perpetuating the cycle of contamination and degradation [8,34].

The use of essential oils represents a promising avenue for mitigating the detrimental effects of microfungi on historical structures. These natural compounds offer a sustainable and environmentally friendly alternative to synthetic biocides, which, while effective, can have adverse effects on the structural integrity of historical materials, human health, and the environment. Synthetic chemicals may also disrupt non-target beneficial organisms, soil microflora, and soil fertility, further emphasizing the need for safer alternatives [17,35].

This study focuses on evaluating the antifungal effects of essential oils derived from plants in the Lamiaceae family. Specifically, the research investigates the impact of these oils on the growth and development of black microcolonial fungi belonging to the genera *Coniosporium*, *Sarcinomyces*, and *Phaeococcomyces*, which are significant contributors to the biodeterioration of historical artifacts [8,33,36,37]. The primary objectives of this study are twofold: first, to assess the efficacy of essential oils in inhibiting the growth of these fungi, thereby protecting historical structures from further fungal-induced deterioration; and second, to promote the use of natural essential oils as sustainable alternatives to synthetic chemicals in the preservation of cultural heritage.

By exploring the antifungal efficacy of essential oils from Lamiaceae plants, this research contributes to the development of safer and more sustainable methods for protecting historical artifacts against fungal degradation. Furthermore, it underscores the importance of integrating natural compounds into conservation practices, thereby promoting the preservation of both cultural heritage and natural ecosystems.

## 2. Materials and methods

This study aimed to evaluate the antifungal properties of essential oils extracted from *Origanum majorana*, *Origanum minutiflorum*, and *Mentha longifolia* subsp. *typhoides* on historical artifacts affected by black microcolonial fungi. The research focused on fungal species such as *Coniosporium*, *Sarcinomyces*, and *Phaeococcomyces*, which are known to contribute to the biodeterioration of historical structures in the Ancient City of Side (Antalya, Türkiye).

Essential oils were extracted from the plants using a Clevenger apparatus and tested *in vitro* against fungal isolates. The oils were applied at three concentrations (0.1%, 1%, and 3%) to assess their antifungal efficacy. Fungal cell counts were conducted before and after treatment to evaluate the inhibitory effects of the essential oils. Statistical analyses were performed to determine significant differences in antifungal activity among the tested concentrations.

### 2.1. Extraction of essential oils

The plants collected from Antalya Ahmetler Canyon and Saklıkent were dried and stored in sterile glass jars for later use. For the identification of plant species, the following references were consulted: Flora of Turkey by Davis [24]; Davis et al. [38]; “A Study on the Flora and Vegetation of Melik and Kaldırım Mountains and Their Surroundings (Manavgat-İbradı/Antalya)” by Çinbilgel [39].

**Fig 1:**
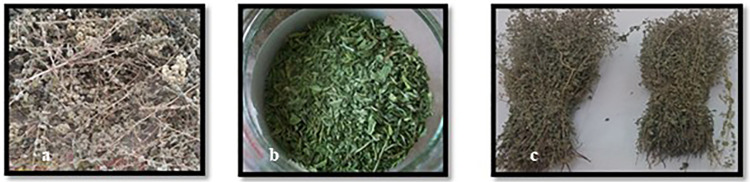
a. Drying of *O. majorana* before extraction. b. *M. longifolia* (dried). c. *O. minutiflorum (*dried).

**Fig 2:**
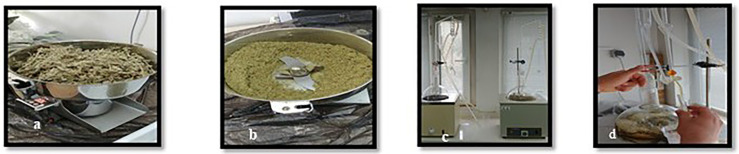
a. b Powdering of *O. majorana,* c. Clevenger Device (double), d. Essential oil from *O. majorana.*

The plants were collected during different seasons, corresponding to their developmental stages, and dried under controlled conditions (Fig 1). The dried plant materials were mechanically ground into smaller pieces using a laboratory grinder. Essential oils were extracted using a Clevenger apparatus through hydrodistillation. The extracted oils were collected in sterile glass tubes, labeled with the plant species and extraction date, and stored at 4°C in a refrigerator until further use (Fig 2).

### 2.2. Isolation and cultivation of microfungi

Corroded stone samples were collected from historical monuments in the Ancient City of Side. The samples were carefully removed using a hammer and scalpel and placed in labeled envelopes indicating the date and location of collection. In the laboratory, the stone particles were washed with 70% ethanol to remove surface contaminants and examined under a stereomicroscope. Black microfungal colonies were isolated using sterile cannulated needles and inoculated onto dichloran rose bengal agar (DRBC, MERCK, Darmstadt, Germany).

**Fig 3:**
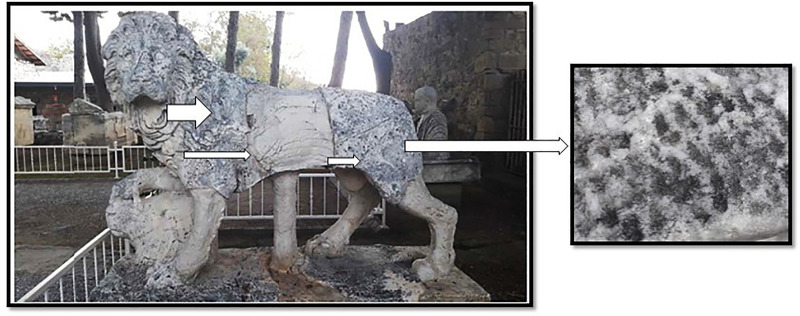
Monuments heavily infected by black microfungi in Side Antique City a. Lion statute b. Column title c. Colonies of microcolonial fungi on marble floor of column title.

As the fungal colonies developed, they were transferred to malt extract agar (MEA, MERCK, Darmstadt, Germany) to obtain pure cultures. Final cultures were grown on MEA and Czapek agar (CzA, MERCK, Darmstadt, Germany) media to ensure the absence of contamination. Microfungal species were identified based on macroscopic and microscopic morphological characteristics, cross-referenced with the molecularly confirmed identifications from Sert et al. (2007) [Ref. 34: Sert H, Sümbül H, Sterflinger K. Microcolonial fungi from antique marbles in Perge/Side/Termessos (Antalya/Turkey). Antonie van Leeuwenhoek. 2007;91:217–227]. This prior study, co-authored by one of the present manuscript’s authors, utilized molecular genetic methods for species identification at the identical archaeological sites and on the same marble substrata as our current work. Our observed morphotypes were directly compared and matched with these established molecularly characterized taxa, ensuring robust and consistent species assignments. Additionally, the fungal identification was supported by the taxonomic keys and descriptions provided in Dematiaceous Hyphomycetes and More Dematiaceous Hyphomycetes [40,41] (Fig 3a–d).

### 2.3. TTC (Triphenyl Tetrazolium Chloride) application

**Fig 4:**
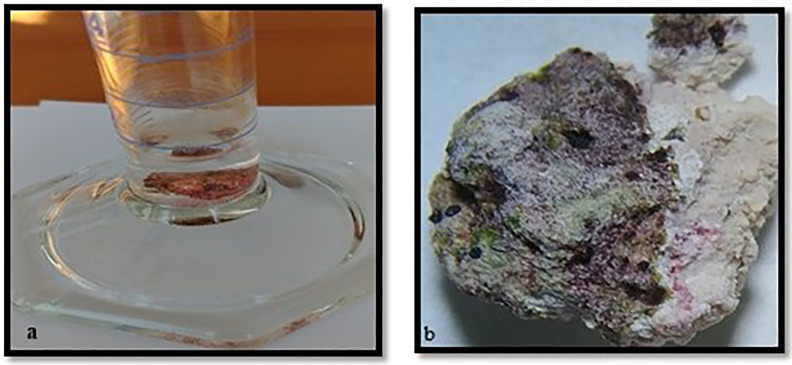
TTC application a. Stone sample in TTC solution b. Observation at stereo microscope after applicatioon.

To assess fungal viability, stone particles infected with black microfungi were treated with essential oil solutions at concentrations of 0.1%, 1%, and 3%. The solutions were prepared with distilled water and applied directly to the stone particles using a spraying method. For the control group, untreated areas of the same samples were subjected to the same culturing and TTC procedures. After treatment, the stone particles were incubated in tubes containing 0.2% TTC solution at 28°C for 24–48 hours. The tubes were wrapped in aluminum foil to prevent light exposure. Following incubation, areas that turned red indicated the presence of viable fungal cells. The treated stone particles were examined under a microscope to observe the development of fungal hyphae in detail (Fig 4).

### 2.4. Collection and counting of spores

Fungal colonies approximately 3 mm in diameter were scraped using a sterile scalpel and transferred to sterile tubes containing 1% Tween 80 solution(polysorbate 80, solubilizer). The suspension was homogenized using a vortex mixer, and spores were collected. The collected spores were counted using a Thoma counting chamber, and a suspension was prepared to contain 10^6 spores/mL in each 1 mm^3^.

One milliliter of the spore suspension was spread onto the surface of MEA plates using a sterile Drigalski spatula. Subsequently, 50 µL of essential oil solutions at different concentrations (0.1%, 1%, and 3%) were pipetted onto the medium. The plates were incubated at 28°C for 48 hours. For the control group, spore suspensions prepared with Tween 80 solution but without essential oils were plated. After incubation, fungal growth was evaluated by counting cells on Thoma slides using conventional colony counting methods in microbiology. All trials were carried out in triplicates.

### 2.5. Statistical analysis

The data were analyzed using the Duncan Multiple Range Test to compare the means of antifungal activity across different concentrations of essential oils. Descriptive statistics were presented as mean and standard deviation values. The assumption of normality was evaluated using the Shapiro-Wilk test, and the assumption of homogeneity of variances was assessed with Levene’s test. For the analysis of differences among more than two groups of numerical data, one-way analysis of variance (ANOVA) was performed. In cases where significant differences were found, pairwise comparisons were conducted using Tukey’s HSD test. All analyses were performed using SAS 9.4 software. A p-value of <0.05 was considered statistically significant.

## 3. Results and discussion

This study investigated the antifungal effects of essential oils extracted from *Origanum majorana*, *Origanum minutiflorum*, and *Mentha longifolia* subsp. *typhoides* on black microcolonial fungi isolated from historical monuments in the Ancient City of Side. The fungal species *Coniosporium*, *Sarcinomyces*, and *Phaeococcomyces* were cultured on malt extract agar (MEA) and Czapek agar (CzA) media, and the effects of essential oils at concentrations of 0.1%, 1%, and 3% were evaluated. The results demonstrated significant antifungal activity, with higher concentrations of essential oils showing greater inhibitory effects.

### 3.1. General observations and TTC test results

The application of essential oils to stone samples infected with black microfungi revealed varying levels of fungal vitality. Samples exhibiting vitality turned red after the TTC (Triphenyl Tetrazolium Chloride) test, as TTC acts as a vital dye that is reduced to red-colored formazan in metabolically active cells. This color change provided a clear indication of fungal viability and allowed for the assessment of the antifungal effects of the essential oils.

**Fig 5:**
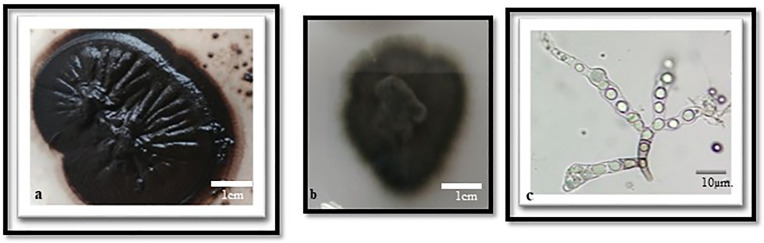
*Phaeococcomyces* sp. (1 month development) a. on MeA, b. on CzA c. Conidiophore.

**Fig 6:**
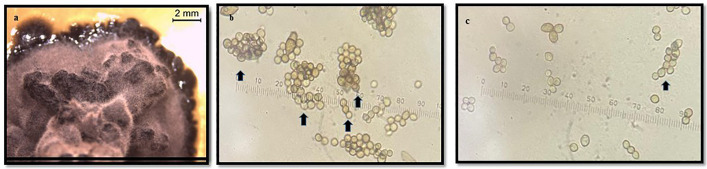
a. *Coniosporium* sp. 1 month development (MeA) b. 2 month development (CzA); c. 1 month development (DRBC).

**Fig 7:**
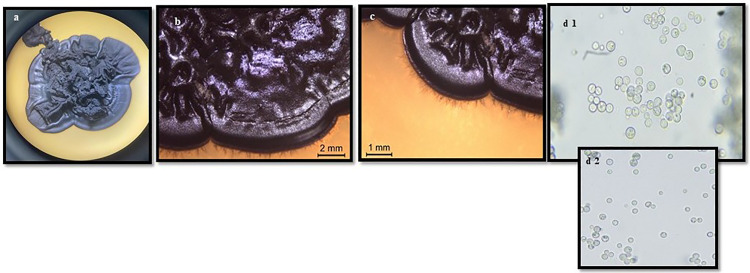
a. *Coniosporium* sp. a. Conidiophores (40x), b. Conidiospores (100x).

**Fig 8:**
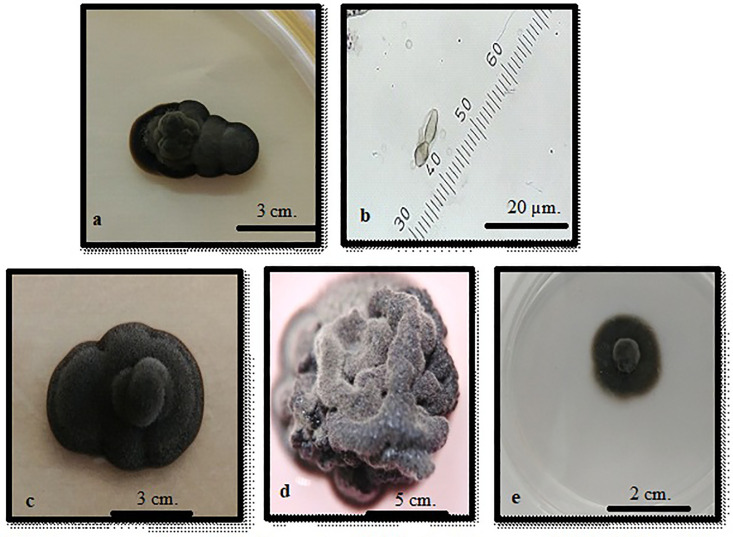
*Sarcinomyces* sp. (1 month development) a. MeA, b. Conidiospore c. MeA d. DRBC e. CzA.

Microscopic and macroscopic observations of black microcolonial fungi grown in the laboratory are presented in Figs 5–8. The spore counts of *Coniosporium*, *Sarcinomyces*, and *Phaeococcomyces* species before and after treatment with essential oils are detailed in Tables 1–6. Statistical analyses, including the Duncan Multiple Range Test, revealed significant differences in fungal growth inhibition across the tested concentrations.

**Table 1:** Spore numbers of *Phaeococcomyces* species before and after the applications A. HA 1016, B. HA 1023, C. HA 1024.

Before the applications	Tween 80 application (Control group)	Concentrations of oils	*Origanum majorana*	*Origanum minutiflorum*	*Mentha longifolia*
674x10^3^ **A**	652x10^3^	0,1%	553x10^3^	592x10^3^	520x10^3^
		1%	400x10^3^	426x10^3^	350x10^3^
		3%	322x10^3^	300x10^3^	295x10^3^
**Before the applications**	**Tween 80 application** **(Control group**	***Concentrations of oils***	***Origanum majorana***	***Origanum minutiflorum***	***Mentha longifolia***
674x10^3^ **B**	668x10^3^	0,1%	616x10^3^	640x10^3^	670x10^3^
		1%	450x10^3^	316x10^3^	534x10^3^
		3%	224x10^3^	212x10^3^	280x10^3^
**Before the applications**	**Tween 80 application** **(Control group**	***Concentrations of oils***	***Origanum majorana***	***Origanum minutiflorum***	***Mentha*** ***Longifolia***
696x103 **C**	688x10^3^	0,1%	636x10^3^	666x10^3^	516x10^3^
		1%	500x10^3^	440x10^3^	465x10^3^
		3%	305x1^03^	310x10^3^	264x10^3^

**Table 2:** Variance analysis and Duncan’s multiple range test results of *Phaeococcomyces* species (The same letter space icons are not significantly different).

Source of change	DF		Tip III SS	Average square	F Value	Pr > F
**Essence**	9		18400,557	2044,5063	6,21	<.0001
**Source**	Sd		KT	KO	F	P
**Model**	9		18400,557	2044,5063	6,21	<.0001
**Tolerance**	90		29639,411	329,32679		
**Adjusted total**	99		48039,968			
**Duncan Grouping**			**Meaning**	**N**	**Essence**	
	A		48.133	10	9	
	A					
	A		46.839	10	6	
	A					
B	A		39.900	10	3	
B	A					
B	A		38.565	10	8	
B	A					
B	A	C	34.976	10	5	
B		C				
B	D	C	28.215	10	2	
	D	C				
E	D	C	19.999	10	4	
E	D	C				
E	D	C	19.681	10	7	
E	D					
E	D		15.427	10	1	
E						
E			5.294	10	10	

**Table 3:** Number of spores of *Coniosporium* sp. before and after the applications A. HA 1001, B. HA 1002, C. HA1003, D. HA1004 E. HA 1005, F. HA 1013, G. HA 1015, H. HA 1018, İ. HA 1019, J. HA 1025.

Before the applications	Tween 80 application (Control group)	Concentrations of oils	*Origanum majorana*	*Origanum minutiflorum*	*Mentha longifolia*
748x10^3^ (1 cm^2^) **A.**		0,1%	722x10^3^	616x10^3^	650x10^3^
	730x10^3^	1%	476x10^3^	504x10^3^	668x10^3^
		3%	414x10^3^	410x10^3^	600x10^3^
**Before the applications**	**Tween 80 application** **(Control group)**	**Concentrations of oils**	***Origanum majorana***	***Origanum minutiflorum***	***Mentha longifolia***
672x10^3^ **B.**	666x10^3^	0,1%	510x10^3^	408x10^3^	526x10^3^
		1%	410x10^3^	366x10^3^	448x10^3^
		3%	304x10^3^	330x10^3^	370x10^3^
**Before the applications**	**Tween 80 application** **(Control group)**	**Concentrations of oils**	***Origanum majorana***	***Origanum minutiflorum***	***Mentha longifolia***
640x103 **C.**	652x10^3^	0,1%	656x10^3^	648x10^3^	638x10^3^
		1%	630x10^3^	632x10^3^	620x10^3^
		3%	554x10^3^	500x10^3^	608x10^3^
**Before the applications**	**Tween 80 application** **(Control group)**	**Concentrations of oils**	***Origanum majorana***	***Origanum minutiflorum***	***Mentha longifolia***
998x103 **D.**	996x10^3^	0,1%	738x10^3^	994x10^3^	868x10^3^
		1%	606x10^3^	390x10^3^	746x10^3^
		3%	578x10^3^	376x10^3^	410x10^3^
**Before the applications**	**Tween 80 application** **(Control group)**	**Concentrations of oils**	***Origanum majorana***	***Origanum minutiflorum***	***Mentha longifolia***
972x103 **E.**	950x10^3^	0,1%	746x10^3^	518x10^3^	960x10^3^
		1%	700x10^3^	500x10^3^	288x10^3^
		3%	712x10^3^	340x10^3^	218x10^3^
**Before the applications**	**Tween 80 application** **(Control group)**	**Concentrations of oils**	***Origanum majorana***	***Origanum minutiflorum***	***Mentha longifolia***
990x103 **F.**	886x10^3^	0,1%	690x10^3^	582x10^3^	562x10^3^
		1%	576x10^3^	388x10^3^	470x10^3^
		3%	404x10^3^	356x10^3^	362x10^3^
**Before the applications**	**Tween 80 application** **(Control group)**	**Concentrations of oil*s***	***Origanum majorana***	***Origanum minutiflorum***	***Mentha longifolia***
880x103 **G.**	765x10^3^	0,1%	728x10^3^	720x10^3^	520x10^3^
		1%	560x10^3^	640x103	170x10^3^
		3%	280x10^3^	600x103	164x10^3^
**Before the applications**	**Tween 80 application** **(Control group)**	**Concentrations of oils**	***Origanum majorana***	***Origanum minutiflorum***	***Mentha longifolia***
720x103 **H.**	600x10^3^	0,1%	665x10^3^	680x10^3^	582x10^3^
		1%	540x10^3^	655x10^3^	467x10^3^
		=	460x10^3^	540x10^3^	354x10^3^
**Before the applications**	**Tween 80 application** **(Control group)**	**Concentrations of oils**	***Origanum majorana***	***Origanum minutiflorum***	***Mentha longifolia***
920x103 **İ.**	852x10^3^	0,1%	712x10^3^	666x10^3^	602x10^3^
		1%	690x10^3^	542x10^3^	360x10^3^
		3%	586x10^3^	366x10^3^	350x10^3^
**Before the applications**	**Tween 80 application** **(Control group)**	**Concentrations of oils**	***Origanum majorana***	***Origanum minutiflorum***	***Mentha longifolia***
776x10^3^ **J.**	765x10^3^	0,1%	760x10^3^	740x10^3^	702x10^3^
		1%	700x10^3^	600x10^3^	670x10^3^
		3%	640x10^3^	450x10^3^	640x10^3^

**Table 4:** Variance analysis and Duncan’s multiple range test results of *Conisporium* species.

Source of change	DF	Tip III SS	Average square	F Value	Pr > F
**Essence**	**9**	**18400,56**	**2044,50631**	**6,21**	**<.0001**
**Source**	**Sd**	**KT**	**KO**	**F**	**P**
**Model**	9	18400,557	2044,50631	6,21	<.0001
**Tolerance**	90	29639,411	329,32679		
Adjusted total	99	48039,968			
**Duncan Grouping**			**Meaning**	**N**	**Essence**
	A		48.133	10	9
	A				
	A		46.839	10	6
	A				
B	A		39.900	10	3
B	A				
B	A		38.565	10	8
B	A				
B	A	C	34.976	10	5
B		C			
B	D	C	28.215	10	2
	D	C			
E	D	C	19.999	10	4
E	D	C			
E	D	C	19.681	10	7
E	D				
E	D		15.427	10	1
E					
E			5.294	10	10

**Table 5:** Number of spores of *Sarcinomyces* species before and after the applications A. HA 1006, B. HA 1007, C. HA 1008, D. HA 1009, E. HA 1010, F. HA 1011, G. HA 1012, H. HA 1017, İ. HA 1021.

Before the applications	Tween 80 application (Control group)	Concentrations of oils	*Origanum majorana*	*Origanum minutiflorum*	*Mentha longifolia*
600x103 **A**	605x10^3^	0,1%	490x10^3^	530x10^3^	602x10^3^
		1%	480x10^3^	526x10^3^	458x10^3^
		3%	192x10^3^	370x10^3^	368x10^3^
**Before the applications**	**Tween 80 application** **(Control group)**	**Concentrations of oils**	***Origanum majorana***	***Origanum minutiflorum***	***Mentha longifolia***
600x10^3^ **B**	580x10^3^	0,1%	518x10^3^	574x10^3^	414x10^3^
		1%	406x10^3^	534x10^3^	396x10^3^
		3%	370x10^3^	494x10^3^	344x10^3^
**Before the applications**	**Tween 80 application** **(Control group)**	**Concentrations of oils**	***Origanum majorana***	***Origanum minutiflorum***	***Mentha longifolia***
708x103 **C**	700x10^3^	0,1%	688x10^3^	572x10^3^	642x10^3^
		1%	638x10^3^	586x10^3^	528x10^3^
		3%	624x10^3^	554x10^3^	478x10^3^
**Before the applications**	**Tween 80 application** **(Control group)**	**Concentrations of oils**	***Origanum majorana***	***Origanum minutiflorum***	***Mentha longifolia***
1000x103 **D**	930x10^3^	0,1%	764x10^3^	862x10^3^	784x10^3^
		1%	704x10^3^	708x10^3^	652x10^3^
		3%	680x10^3^	664x10^3^	526x10^3^
**Before the applications**	**Tween 80 application** **(Control group)**	**Concentrations of oils**	***Origanum majorana***	***Origanum minutiflorum***	***Mentha longifolia***
870x103 **E**	660x10^3^	0,1%	526x10^3^	470x10^3^	456x10^3^
		1%	516x10^3^	410x10^3^	416x10^3^
		3%	408x10^3^	322x10^3^	370x10^3^
**Before the applications**	**Tween 80 application** **(Control group)**	**Concentrations of oils**	***Origanum majorana***	***Origanum minutiflorum***	***Mentha longifolia***
690x103**F**	670x10^3^	0,1%	592x10^3^	550x10^3^	582x10^3^
		1%	504x10^3^	494x10^3^	484x10^3^
		3%	456x10^3^	446x10^3^	424x10^3^
**Before the applications**	**Tween 80 application** **(Control group)**	**Concentrations of oils**	***Origanum majorana***	***Origanum minutiflorum***	***Mentha longifolia***
710x103 **G**	674x10^3^	0,1%	634x10^3^	654x10^3^	646x10^3^
		1%	536x10^3^	606x10^3^	518x10^3^
		3%	478x10^3^	554x10^3^	472x10^3^
**Before the applications**	**Tween 80 application** **(Control group)**	**Concentrations of oils**	***Origanum majorana***	***Origanum minutiflorum***	***Mentha longifolia***
916x103 **H**	860x10^3^	0,1%	520x10^3^	594x10^3^	554x10^3^
		1%	506x10^3^	570x10^3^	430x10^3^
		3%	476x10^3^	536x10^3^	376x10^3^
**Before the applications**	**Tween 80 application** **(Control group)**	**Concentrations of oils**	***Origanum majorana***	***Origanum minutiflorum***	***Mentha longifolia***
952x103 **I**	870x10^3^	0,1%	370x10^3^	320x10^3^	980x10^3^
		1%	320x10^3^	296x10^3^	976x10^3^
		3%	230x10^3^	270x10^3^	550x10^3^

**Table 6:** Variance analysis and Duncan’s multiple range test results of *Sarcinomyces* species.

Source of change	DF	Tip III SS	Average square	F Value	Pr > F
**Essence**	**9**	**18400.55677**	**2044.50631**	**6.21**	**<.0001**
**Source**	**Sd**	**KT**	**KO**	**F**	**P**
**Model**	9	18400.55677	2044.50631	6.21	<.0001
**Tolerance**	90	29639.41116	329.32679		
**Adjusted total**	99	48039.96793			
**Duncan Grouping**			**Meaning**	**N**	**Essence**
	A		48.133	10	9
	A				
	A		46.839	10	6
	A				
B	A		39.900	10	3
B	A				
B	A		38.565	10	8
B	A				
B	A	C	34.976	10	5
B		C			
B	D	C	28.215	10	2
	D	C			
E	D	C	19.999	10	4
E	D	C			
E	D	C	19.681	10	7
E	D				
E	D		15.427	10	1
E					
E			5.294	10	10

### 3.2. Essential oil applications on fungi

The slow-growing *Phaeococcomyces* species, which inhabits rocks and forms dark brown to black colonies, exhibited significant sensitivity to essential oil treatments (Fig 5). The number of spores before treatment, after treatment with Tween 80 (1% solution), and after essential oil applications at concentrations of 0.1%, 1%, and 3% are provided in Table 1. Statistical analyses (Table 2) revealed that higher concentrations of essential oils, particularly 3%, resulted in a marked reduction in spore counts.

The pronounced antifungal effect on *Phaeococcomyces* may be attributed to its thinner and more permeable cell walls, which facilitate the penetration of essential oil compounds. This finding aligns with previous studies highlighting the susceptibility of certain fungal species to essential oils due to differences in cell wall composition [17,42].

The *Coniosporium* species, characterized by its black, meristematic thallus and moriform colonies, displayed moderate sensitivity to essential oil treatments. Colonies grown on MEA exhibited a black, dry, and cerebriform texture, while those on CzA were flat with irregular margins. Microscopic observations revealed pale brown to dark brown hyphae with cylindrical to spherical cells and varying cell wall thicknesses (Figs 6 and 7).

Spore counts before and after treatment with Tween 80 (1%) and essential oils at concentrations of 0.1%, 1%, and 3% are presented in Table 3 and variance analysis and Duncan’s multiple range test results of *Conisporium* species presented in Table 4.

Statistical analyses indicated that while all concentrations inhibited fungal growth, the 3% concentration was the most effective. However, the antifungal effect was less pronounced compared to *Phaeococcomyces*, likely due to the thicker and more robust cell walls of *Coniosporium* species.

Colonies of *Sarcinomyces* species grown on MEA appeared dark grey to black, transitioning to a cerebriform texture with age. On CzA, colonies initially appeared black and soft, later developing pale grey aerial mycelium with irregular margins (Fig 8). These morphological characteristics are consistent with previous descriptions of *Sarcinomyces* species [41].

Spore counts before and after treatment with Tween 80 (1%) and essential oils at concentrations of 0.1%, 1%, and 3% are provided in Table 5. Statistical analyses (Table 6) revealed significant growth inhibition at higher concentrations, with the 3% concentration showing the greatest effect. However, the antifungal activity was less pronounced compared to *Phaeococcomyces*, likely due to differences in cell wall structure and composition.

**Fig 9:**
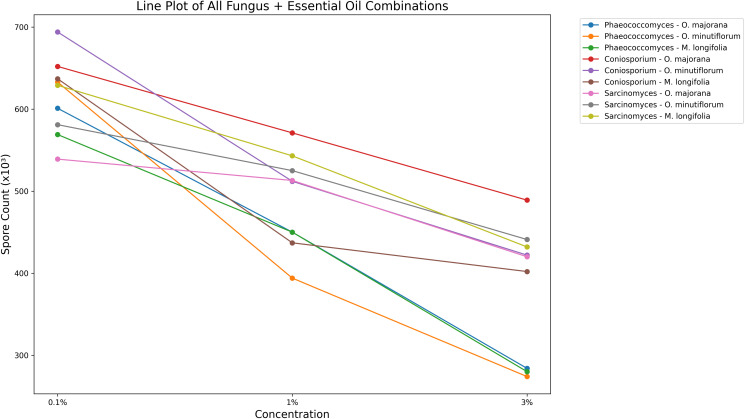
Line plot illustrating the antifungal effects of different essential oils (*O. majorana*, *O. minutiflorum*, *M. longifolia*) at various concentrations (0.1%, 1%, 3%) on species of *Phaeococcomyces, Coniosporium, Sarcinomyces.*

The line plot illustrates the effect of increasing concentrations (0.1%, 1%, 3%) of three different essential oils (*O. majorana, O. minutiflorum, M. longifolia*) on the spore counts of fungal species of *Phaeococcomyces*, *Coniosporium*, *Sarcinomyces (*Fig 9). In all fungal species, a marked decrease in spore count was observed as the essential oil concentration increased. Notably, *O. minutiflorum* at 3% concentration exhibited the highest antifungal activity, particularly against *Phaeococcomyces* and *Coniosporium*. These results demonstrate that the antifungal efficacy of essential oils is concentration-dependent and that certain combinations are more effective than others.

**Fig 10:**
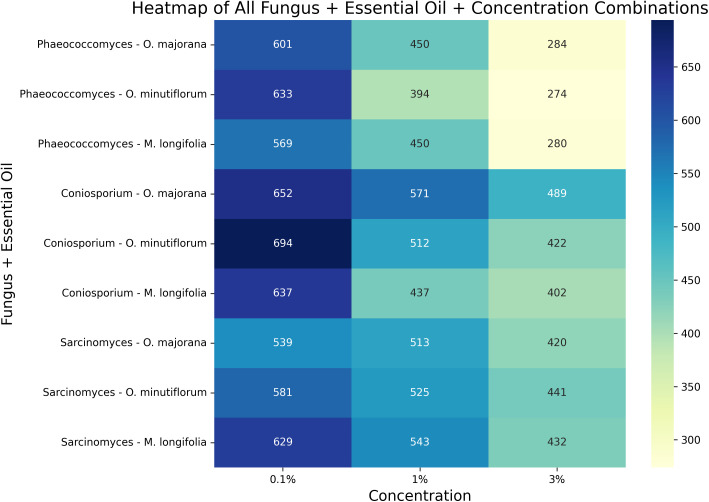
Heatmap illustrating the spore counts of different fungal species and essential oil combinations at various concentrations (0.1%, 1%, 3%). Darker colors represent lower spore counts (higher antifungal efficacy), while lighter colors indicate higher spore counts (lower antifungal efficacy).

The heatmap provides a visual summary of spore count variations across all fungal species and essential oil combinations at different concentrations (Fig 10). The lowest spore counts are indicated by darker colors, while higher counts are shown in lighter shades. Notably, *O. minutiflorum* at 3% concentration resulted in the lowest spore counts for *Phaeococcomyces* and *Coniosporium*. This visualization allows for the rapid identification of the most effective combinations and clearly demonstrates the potential of essential oils against biodeteriogenic fungi.

Table 7 shows the results of the normality test (Shapiro-Wilk) and Levene’s test for each fungal genus at each essential oil dose. This table evaluates the normality (Shapiro-Wilk test) and homogeneity of variances (Levene’s test) of the differences obtained before and after the application of various essential oil doses for three different fungal genera (*Coniosporium, Sarcinomyces,* and *Phaeococcomyces*). Since the assumption of normality (p > 0.05) and homogeneity of variances (p > 0.05) were met for all three fungal species, the use of parametric methods appears to be appropriate.

**Table 7:** The results of the normality test (Shapiro-Wilk) and Levene’s test for each fungal genus at each essential oil dose.

			Shapiro Wilk	Levene Test
			p	P
Differences (Before-After)	Coniosporium	O. majorana %0.1	0,451	0,206
		O. majorana %1	0,622	
		O. majorana % 3	0,623	
		O. minutiflorum %0.1	0,245	
		O.minutiflorum %1	0,678	
		O.minutiflorum %3	0,122	
		M.longifolia %0,1	0,178	
		M. longifolia %1	0,217	
		M.longifolia %3	0,337	
		Control	0,050	
Differences (Before-After)	Sarcinomyces	O. majorana %0.1	0,150	0,255
		O. majorana %1	0,339	
		O. majorana % 3	0,559	
		O. minutiflorum %0.1	0,061	
		O.minutiflorum %1	0,183	
		O.minutiflorum %3	0,273	
		M.longifolia %0,1	0,407	
		M. longifolia %1	0,434	
		M.longifolia %3	0,193	
		Control	0,053	
Differences (Before-After)	Phaeococcomyces	O. majorana %0.1	0,053	0,083
		O. majorana %1	0,060	
		O. majorana % 3	0,865	
		O. minutiflorum %0.1	0,132	
		O.minutiflorum %1	0,125	
		O.minutiflorum %3	0,241	
		M.longifolia %0,1	0,262	
		M. longifolia %1	0,988	
		M.longifolia %3	0,531	
		Control	0,220	

**Fig 11:**
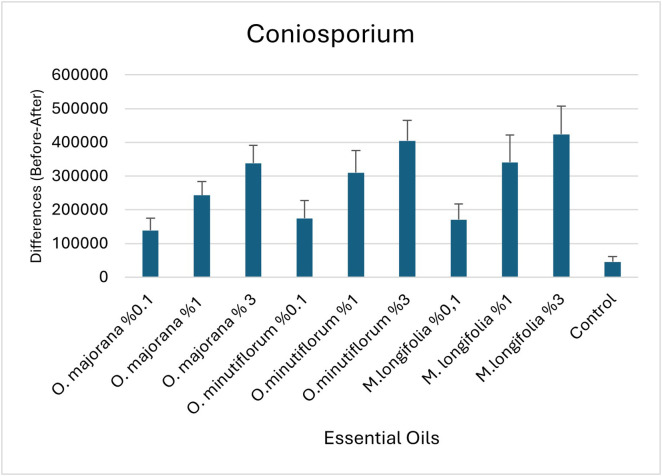
Bar graphs (with standard errors) of essential oil concentrations in terms of the difference (Before-After) in cell count for the genus *Coniosporium.*

Comparison of essential oil concentrations in terms of the difference (Before-After) in cell count for the genus *Coniosporium* are specified in Table 8 and bar graphs are shown in Fig 11. This table compares the effects of different types and doses of essential oils on the difference in cell count before and after essential oil application in the genus *Coniosporium*. According to the ANOVA results, there are statistically significant differences in cell count changes among the essential oil treatments (p < 0.0001). This indicates that the different types and concentrations of oils applied have a significant effect on *Coniosporium*.

**Table 8:** Comparison of essential oil concentrations in terms of the difference (Before-After) in cell count for the genus *Coniosporium.*

Esansiyel Yağ	N	Mean	Standard Deviation	p	Tukey’s HSD	Effect size (partial eta squared)
O. majorana %0.1	10	138900	111837,63	<0,0001	bc	0,325
O. majorana %1	10	242800	127248,49		abc	
O. majorana % 3	10	338400	168086,75		ab	
O. minutiflorum %0.1	10	174400	166212,18		abc	
O.minutiflorum %1	10	309900	206395,76		ab	
O.minutiflorum %3	10	404800	189863,58		A	
M.longifolia %0,1	10	170600	147328,96		abc	
M. longifolia %1	10	340900	255941,16		ab	
M.longifolia %3	10	424000	261411,55		A	
Control	10	45400	51171,17		C	

Different letters within a column indicate a statistically significant difference between essential oil doses (p < 0.05).

The Tukey HSD multiple comparison test indicated group differences with letters:

The lowest effect was observed in the control group (c). The highest difference was found in the *M. longifolia* 3% and *O. minutiflorum* 3% groups (a). These groups are statistically different from the control group.

Moderate effects were observed in groups such as *O. majorana* 3%, *O. minutiflorum* 1%, *M. longifolia* 1%, and *O. majorana* 1%, which are included in groupings like ab and abc.

η² = 0.325, meaning that 32.5% of the variance can be attributed to the type and dose of essential oil applied.

This can be considered a medium to large effect size (according to Cohen [43]: 0.01 = small, 0.06 = medium, 0.14 = large).

**Fig 12:**
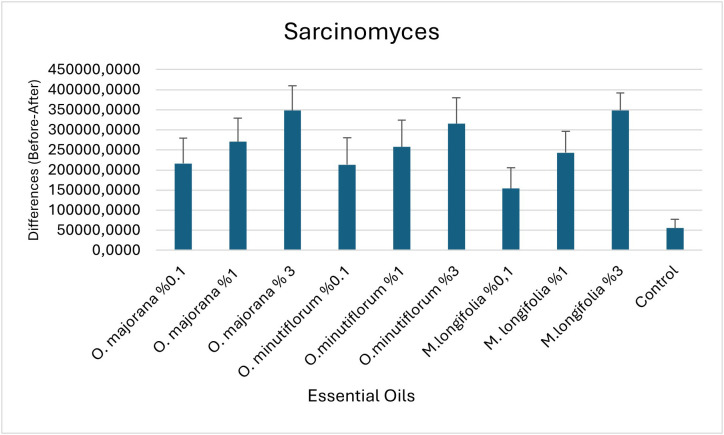
Bar graphs (with standard errors) of essential oil concentrations in terms of the difference (Before-After) in cell count for the genus *Sarcinomyces.*

Comparison of essential oil concentrations in terms of the difference (Before-After) in cell count for the genus *Sarcinomyces* are shown in Table 9; bar graphs are presented in Fig 12.

**Table 9:** Comparison of essential oil concentrations in terms of the difference (Before-After) in cell count for the genus *Sarcinomyces.*

Esansiyel Yağ	N	Mean	Standard Deviation	p	Tukey’s HSD	Effect size (partial eta squared)
O. majorana %0.1	10	216000,0000	188504,64185	0,013	ab	0,222
O. majorana %1	10	270666,6667	174255,55945		ab	
O. majorana % 3	10	348000,0000	185671,75337		a	
O. minutiflorum %0.1	10	213333,3333	199799,89990		ab	
O.minutiflorum %1	10	257333,3333	201519,22985		ab	
O.minutiflorum %3	10	315111,1111	193904,90224		ab	
M.longifolia %0,1	10	154000,0000	154576,84173		ab	
M. longifolia %1	10	243111,1111	160290,70812		ab	
M.longifolia %3	10	348666,6667	129143,33123		a	
Control	10	55222,2222	64806,97836		b	

Different letters within a column indicate a statistically significant difference between essential oil doses (p < 0.05).

ANOVA analysis showed that there are statistically significant differences in the change in *Sarcinomyces* cell count among different types and doses of essential oils (p = 0.013). This indicates that the treatments applied have a significant effect on fungal cell counts.

According to the results of the Tukey HSD test: The lowest effect was observed in the control group (b), which is statistically different from the others. The highest effect was obtained with *O. majorana* 3% and *M. longifolia* 3% applications (group a); there is no statistical difference between these two groups, but both are different from the control group. All other treatments (ab) showed values close to both the high-dose groups and the control group, and therefore statistically overlap with both group a and group b.

An η² value of 0.222 indicates that the effect of the treatment is between medium and large (According to [43] — 0.01 = small, 0.06 = medium, 0.14 = large effect).

**Fig 13:**
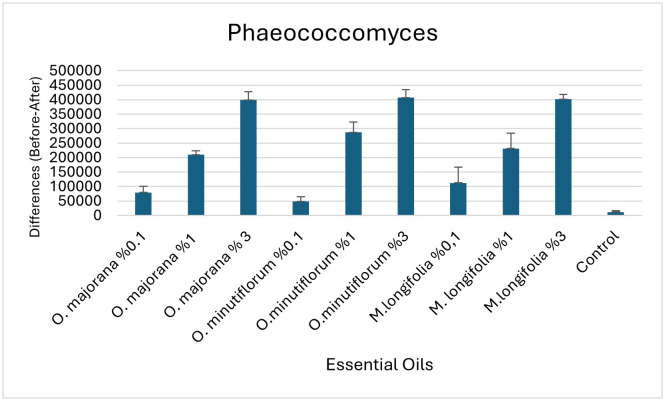
Bar graphs (with standard errors) of essential oil concentrations in terms of the difference (Before-After) in cell count for the genus *Phaeococcomyces.*

Comparison of essential oil concentrations in terms of the difference (Before-After) in cell count for the genus *Phaeococcomyces* are specified in Table 10 and bar graphs are shown in Fig 13.

**Table 10:** Comparison of essential oil concentrations in terms of the difference (Before-After) in cell count for the genus *Phaeococcomyces.*

Esansiyel Yağ	N	Mean	Standard Deviation	p	Tukey’s HSD	Effect size (partial eta squared)
O. majorana %0.1	10	79666,67	35809,68	<0,0001	cde	0,915
O. majorana %1	10	210000,00	19798,99		bcd	
O. majorana % 3	10	399000,00	49122,30		a	
O. minutiflorum %0.1	10	48666,67	28936,71		de	
O.minutiflorum %1	10	287333,33	61329,71		ab	
O.minutiflorum %3	10	407333,33	47721,41		a	
M.longifolia %0,1	10	112666,67	95001,75		cde	
M. longifolia %1	10	231666,67	92001,81		bc	
M.longifolia %3	10	401666,67	27319,10		a	
Control	10	12000,00	8717,80		e	

The results demonstrate that essential oil type and concentration have a highly significant effect on the change in *Phaeococcomyces* cell count (p < 0.0001).

The results demonstrate that essential oil type and concentration have a highly significant effect on the change in *Phaeococcomyces* cell count (p < 0.0001).

Tukey HSD test:

- The greatest antifungal effect was observed with *O. minutiflorum* 3%, *M. longifolia* 3%, and *O. majorana* 3% (group a), which produced the largest cell-count reductions.
- The lowest effect occurred in the control group (group e), which differed significantly from all other groups.
- All remaining treatments fell into various intermediate groups, with the 1% doses generally showing moderate effects (groups ab, bc, cde, etc.).

η² = 0.915 indicates an extremely large effect size, meaning that 91.5% of the variance can be attributed to the essential‐oil type and dose applied. According to Cohen’s classification [43], this represents a very large effect, underscoring the strong biological impact of the treatments.

### 3.5. Broader implications for cultural heritage preservation

Cultural heritage sites are highly susceptible to biodeterioration caused by microorganisms such as algae, mosses, lichens, bacteria and fungi. Traditional chemical biocides, while effective, pose significant risks to the environment and human health. In contrast, essential oils, which are rich in bioactive secondary metabolites, offer a natural and environmentally friendly alternative for controlling biodeteriogenic microorganisms [42,44–47].

The results of this study demonstrated that essential oils derived from *O. majorana*, *O. minutiflorum*, and *M. longifolia* effectively inhibited the growth of black microcolonial fungi. Among the tested oils, *O. minutiflorum* at a 3% concentration exhibited the highest antifungal activity, followed by *M. longifolia*. These findings are consistent with previous research highlighting the antifungal properties of essential oils against biodeteriogenic fungi [17,42].

### 3.6. Practical considerations for essential oil application

While essential oils show great promise for the preservation of historical artifacts, their application must be carefully controlled. Over time, essential oils undergo chemical changes, including increased peroxide content, acidity, and resinification, which can alter their physical properties and potentially damage stone surfaces. Therefore, it is crucial to use essential oils at optimal concentrations tailored to the specific oil and the material being treated.

Recent studies have further highlighted the potential and challenges of using essential oils and related biotechnological approaches in the conservation of cultural heritage. For example, Mohamed et al. [48] investigated a mixture of essential oils (*Origanum vulgare, Moringa oleifera, and Cinnamomum verum*) for controlling fungal deterioration in wall paintings and found that oregano oil exhibited the strongest antifungal effect, with optimized mixtures achieving complete inhibition of all tested fungal strains. Importantly, their study also addressed cytotoxicity and colorimetric changes, noting that while essential oils are effective, some may cause color alterations on sensitive substrates, emphasizing the need for careful selection and testing of oils for conservation purposes [48].

Similarly, the study by Yao et al. According to [49] focused on the enhanced production of fungal laccase enzymes, which are relevant for biotechnological applications in biodeterioration control and cleaning of cultural heritage materials. Their work demonstrates the growing role of enzyme-based and microbial approaches as complementary or alternative strategies to essential oil treatments [49]. However, as also noted in the literature, complete eradication of fungal contamination was not achieved, and potential effects on material properties must be considered. These comparisons underscore the need for further research into optimizing essential oil formulations, evaluating their long-term impacts, and integrating them with other biotechnological methods for effective and sustainable conservation of cultural heritage.

Khaled et al. [50] demonstrated that a mixture of oregano, rosemary, and mint essential oils achieved up to 99.65% inhibition of *Penicillium digitatum* growth in citrus fruits, highlighting the strong antifungal potential of Lamiaceae-derived oils in food preservation. Similarly, our study showed that essential oils from Lamiaceae species exhibited significant antifungal activity against biodeteriogenic black fungi on cultural heritage stones. While both studies emphasize the efficacy and biosafety of essential oils, Khaled et al. focused on postharvest disease control in agriculture, whereas our research addresses the conservation of cultural heritage materials. Together, these findings underscore the broad applicability of plant essential oils as green biocidal agents in both food safety and heritage conservation contexts.In line with these recent findings, our study confirms the significant antifungal activity of Lamiaceae-derived essential oils against black microcolonial fungi on historical monuments.

In addition to essential oils, various alternative methods have been explored for the removal of biological colonization on stone monuments. One such method involves the use of dimethyl sulfoxide (DMSO), as described by Toreno et al. (2018), who developed a low-impact cleaning technique for stone surfaces affected by microbial growth. While DMSO-based treatments have demonstrated effectiveness in reducing microbial colonization, they may present certain limitations, such as potential chemical interactions with stone substrates and the need for careful handling due to their solvent properties. In comparison, plant-derived essential oils offer a more environmentally sound and sustainable approach, with lower toxicity and reduced environmental impact. However, the present study found that although essential oils significantly reduced the number of black microcolonial fungi, complete eradication was not achieved, suggesting that repeated or combined treatments may be necessary for optimal results. Further comparative studies are needed to fully assess the long-term efficacy, material compatibility, and practical applications of essential oils versus other cleaning agents such as DMSO in the conservation of cultural heritage [51].

The findings of this study suggest that essential oils, when used in controlled doses, can provide an effective and environmentally friendly solution for the protection and restoration of cultural heritage sites. Future research should focus on the long-term effects of essential oil treatments and their interactions with different types of stone materials.

## 4. Conclusions

This study highlights the potential of essential oils derived from *Origanum majorana*, *Origanum minutiflorum*, and *Mentha longifolia subsp. typhoides* as effective antifungal agents for the preservation of historical monuments. The results demonstrated that these natural compounds exhibit significant antifungal activity against black microcolonial fungi, including *Coniosporium*, *Sarcinomyces*, and *Phaeococcomyces*, which are major contributors to the biodeterioration of cultural heritage. Among the tested essential oils, *O. minutiflorum* at a 3% concentration showed the highest inhibitory effect, followed by *M. longifolia*.

The findings underscore the importance of eco-friendly and sustainable alternatives to synthetic biocides in the conservation of historical artifacts. Essential oils not only provide an tabenvironmentally safe solution but also align with the growing demand for sustainable preservation methods that minimize harm to both cultural heritage and the surrounding ecosystem.

Future research should focus on the long-term efficacy of essential oil treatments under real-world conditions, including their interactions with different types of stone materials and environmental factors. Although this study focused on the antifungal efficacy of essential oils. In restoration applications, it is also important to consider the interactions between biocides and the substrate. In recent years, standardized approaches and comprehensive analyses have been developed to assess the potential impacts of biocides on stone materials [31]. Future studies should investigate the long-term effects and possible side effects of essential oils on stone surfaces in detail. Additionally, further studies are needed to optimize application methods and concentrations to ensure maximum effectiveness while preventing potential adverse effects on the structural integrity of historical materials.

By integrating natural compounds such as essential oils into conservation practices, this study contributes to the development of innovative and sustainable strategies for protecting cultural heritage from biodeterioration, ensuring its preservation for future generations.
